# Congenital Atresia of the Esophagus

**Published:** 1913-05

**Authors:** 


Congenital Atresia of the Esophagus. *Archiv für Kinderheilkunde.* W. P. Shukowsky and A. H. Bacon report the case of an infant that manifested a slight degree of asphyxia at birth and that was not able to perform the act of sucking. There was a persistent discharge from the nose, of a tenacious grayish white section. Death occurred in forty-eight hours. At autopsy there was found a dilatation of the esophagus that terminated below in a blind pouch. This communicated by means of a slitlike fistula with the trachea.

